# Impact of the Mediterranean Diet on Patients With Psoriasis: Protocol for a Randomized Controlled Trial

**DOI:** 10.2196/64277

**Published:** 2025-01-29

**Authors:** Javier Perez-Bootello, Emilio Berna-Rico, Carlota Abbad-Jaime de Aragon, Ruth Cova-Martin, Leticia Goni, Asuncion Ballester-Martinez, Pedro Jaen-Olasolo, Nehal Mehta, Joel M Gelfand, Miguel Angel Martinez-Gonzalez, Alvaro Gonzalez-Cantero

**Affiliations:** 1 Department of Dermatology Hospital Universitario Ramon y Cajal, Instituto Ramón y Cajal de Investigación Sanitaria (IRYCIS), Madrid, Spain Madrid Spain; 2 Department of Preventive Medicine and Public Health, IdiSNA (Instituto de Investigación Sanitaria de Navarra), University of Navarra, Pamplona IdiSNA (Instituto de Investigación Sanitaria de Navarra), University of Navarra Pamplona Spain; 3 CIBER Fisiopatología de la Obesidad y Nutrición (CIBERObn), Instituto de Salud Carlos III Madrid Spain; 4 National Heart, Lung, and Blood Institute, National Institutes of Health, Bethesda, Maryland Department of Cardiology, George Washington Medical Center, Washington, District of Columbia, USA Bethesda, MD United States; 5 Department of Dermatology, University of Pennsylvania Perelman School of Medicine, Philadelphia, Pa Philadelphia, PA United States; 6 Department of Biostatistics, Epidemiology and Informatics, University of Pennsylvania Perelman School of Medicine Philadelphia, PA United States; 7 Department of Preventive Medicine and Public Health, IdiSNA (Instituto de Investigación Sanitaria de Navarra), University of Navarra, Pamplona Pamplona Spain; 8 Department of Nutrition, Harvard T.H. Chan School of Public Health Boston, MA United States; 9 Faculty of Medicine, Universidad Francisco de Vitoria Madrid Spain

**Keywords:** psoriasis, Mediterranean diet, MedDiet, nutritional intervention, olive oil, inflammatory disease, Impact of the Mediterranean Diet on Patients with Psoriasis, MEDIPSO, dietary intervention, methodological analysis, randomized controlled trial, RCT, clinical trial, nutrition, diet, Europe

## Abstract

**Background:**

Psoriasis is an inflammatory disease primarily treated through molecular-targeted therapies. However, emerging evidence suggests that dietary interventions may also play a role in managing inflammation associated with this condition. The Mediterranean diet (MedDiet), prevalent in southern European countries, has been widely recognized for its ability to reduce cardiovascular mortality, largely due to its anti-inflammatory properties. This anti-inflammatory potential has prompted interest in exploring the MedDiet’s role in immune-mediated diseases, including psoriasis. Observational studies have indicated potential benefits, such as reductions in the Psoriasis Area and Severity Index. However, there is a need for well-designed clinical trials to address the methodological limitations of these studies and to establish specific dietary recommendations for psoriasis.

**Objective:**

This study aims to evaluate the impact of an intensive dietary intervention based on the MedDiet in patients with psoriasis. The study will assess the effects of this intervention on skin involvement, metabolic parameters, and inflammatory cytokines. In addition, the emotional well-being and quality of life of participants will be evaluated using validated questionnaires. A methodological analysis will also be conducted to enhance the design of future large-scale clinical trials.

**Methods:**

An open-label, single-blinded (evaluator) randomized controlled trial was designed to assess the impact of a high-intensity MedDiet intervention in patients with mild-to-moderate psoriasis. A total of 38 patients will be randomly assigned into 2 groups—an intervention group receiving the MedDiet intervention and a control group receiving standard care. The intervention group will participate in dietary education sessions aimed at adopting the MedDiet over 4 months, with monthly monitoring by experienced nutritionists. Participants will receive 500 mL of extra virgin olive oil per week, along with informative materials, recipes, and weekly menus. In contrast, the control group will receive standard low-fat diet recommendations without nutritionist monitoring. All participants will undergo a baseline visit, a 2-month follow-up visit, and a final visit at 4 months. Blood tests will be conducted at the beginning and end of the study. This study protocol was approved by the Institutional Review Board of the Hospital Ramón y Cajal (Madrid) in July 2023.

**Results:**

Enrollment concluded in October 2024, with data collection set to finish by February 2025. The findings will be presented at national and international conferences and published in peer-reviewed journals.

**Conclusions:**

This protocol outlines the design of a clinical trial that implements the MedDiet in patients with psoriasis to evaluate its benefits on skin involvement, systemic inflammation, and quality of life.

**Trial Registration:**

ClinicalTrials.gov NCT06257641; https://clinicaltrials.gov/study/NCT06257641

**International Registered Report Identifier (IRRID):**

DERR1-10.2196/64277

## Introduction

Psoriasis is a common skin disease, affecting between 2% and 4% of the population [1,2]. Its pathogenesis involves a complex interplay between innate and adaptive immunity, with the interleukin (IL)-17/IL-23 axis playing a crucial role. While not all underlying molecular mechanisms have been fully elucidated, current medical treatments primarily target proinflammatory cytokines [3]. In today’s era of media, nutrition has become a significant topic of interest among patients with psoriasis. A survey conducted by the National Psoriasis Foundation involving 1206 patients revealed that 89% (n=1073) believed it was important to discuss the role of diet in their disease with their physician, and 86% (n=1037) had attempted dietary modifications. Despite this, only 31% (374/1206) of patients had actually discussed dietary changes with a health care provider [4]. In fact, nutritional approaches play a very important role in the prevention and treatment process of chronic diseases such as inflammatory diseases, autoimmune diseases, cardiovascular diseases, and even mental diseases [5,6].

There is well-established evidence supporting the beneficial effect of weight loss in patients with psoriasis [7]. Dietary interventions that promote weight loss are recognized as valuable therapeutic strategies for reducing inflammation, particularly in obese patients with psoriasis [8]. In addition, the nutrigenomic effects of certain food components have been documented [9]. However, the only firm dietary recommendations currently reflected in most psoriasis guidelines and expert consensus are weight loss for patients who are overweight and obese and a gluten-free diet for those with positive celiac disease serology [10]. Therefore, there remains a need for dietary guidance that offers benefits beyond weight loss, applicable to patients of any body weight.

The Mediterranean diet (MedDiet) refers to the eating patterns typical of the olive-growing regions around the Mediterranean Sea. This dietary approach emphasizes the consumption of fruits, whole grains, vegetables, nuts, seeds, legumes, and olive oil—the hallmark of the MedDiet. It also includes a moderate intake of red wine with meals, seafood, poultry, and fermented dairy products, while limiting red and processed meats, sweets, and other ultraprocessed foods [11]. Growing evidence suggests that the MedDiet may influence the progression and severity of psoriasis [12,13]. In a 2018 cross-sectional study by Phan et al [14], which included 35,735 participants from the NutriNet-Santé cohort, 3557 participants had psoriasis, with 878 classified as severe cases. The multivariate analysis, adjusted for factors such as age, sex, weight, and cardiovascular risk, showed a significantly lower percentage of severe psoriasis cases among those with greater adherence to the MedDiet [14].

Several research groups have designed large-scale dietary interventions to implement the MedDiet in large patient cohorts. The PREDIMED (Prevention with Mediterranean Diet) study [15], which included 7447 patients at high risk of cardiovascular disease, randomly assigned participants into 3 groups—2 groups received Mediterranean dietary advice supplemented with extra virgin olive oil (EVOO) or a mixture of nuts, while the third group followed low-fat dietary recommendations. All groups were closely monitored by nutritionists. The PREDIMED study demonstrated the positive role of the MedDiet in preventing cardiovascular events. Subsequent satellite studies, such as the PREDIMAR (Prevention of Recurrent Arrhythmias with Mediterranean Diet) study [16], which used an entirely online dietary intervention, further underscored the MedDiet’s benefits and the feasibility of achieving high adherence to this dietary pattern through a dietary intervention.

The MEDIPSO (Impact of the Mediterranean Diet on Patients with Psoriasis) study, detailed in this paper, was conceived to address the need for more evidence on the relationship between psoriasis and the MedDiet. This proof-of-concept randomized controlled trial, inspired by the PREDIMED study, involves a high-intensity MedDiet intervention in patients with psoriasis. The primary objective is to evaluate the impact of this dietary pattern on psoriasis skin involvement, inflammatory cytokines, and quality of life.

## Methods

### Study Design

The MEDIPSO study is an open-label, single-blinded (evaluator) randomized controlled trial. This experimental study involves patients with mild-to-moderate psoriasis who are on stable topical treatment, which will be randomly assigned to either a control group or an intervention group. Patients in the intervention group will receive dietary education focused on adopting the MedDiet, with monthly monitoring by nutritionists experienced in the PREDIMED clinical trial. In contrast, the control group will not receive any active dietary modification interventions.

Both groups will undergo blood tests to assess metabolic parameters before and after the study period, and data on anthropometric characteristics and quality of life will be collected. The study aims to evaluate the effects of the MedDiet on skin involvement, metabolic parameters, and inflammatory cytokines, as well as its impact on emotional well-being and quality of life. In addition, a methodological analysis will be conducted to refine the design for future large-scale clinical trials.

### Participants

In total, 38 patients with mild-to-moderate plaque psoriasis and no systemic treatment will be included. Participants for this study will be recruited from among patients who regularly attend dermatology consultations at Hospital Universitario Ramón y Cajal. The inclusion criteria and exclusion criteria are stated in Textbox 1.

**Inclusion and exclusion criteria for the participation in the Impact of the Mediterranean Diet on Patients with Psoriasis study.**

**Inclusion criteria:**

- Psoriasis clinically diagnosed by an experienced dermatologist
- Predominantly psoriasis vulgaris
- Psoriasis Area and Severity Index ≥2 and ≤10 at the time of recruitment
- Stable weight (<5% weight loss or gain) in the last 3 months
- Treated exclusively with topical treatment for psoriasis at enrollment and throughout the study
- Willing and able to follow the study procedure, attend all scheduled visits during the study period, and provide blood samples as indicated in the procedure
- Able to give informed consent
- Willing to implement pregnancy prevention measures throughout the study period

**Exclusion criteria:**

- Type 1 or 2 diabetes mellitus
- Good adherence to the Mediterranean diet at the time of screening (energy-restricted Mediterranean Diet Adherence Screener score ≥8)
- Language barrier (patients not fluent in Spanish or English) or conditions that make telephone communication difficult (eg, severe hearing loss)
- History of cardiac disease
- Comorbidities that may compromise the implementation of the intervention (eg, cancer, digestive diseases, and so on) or limit survival to less than 6 months
- History or current eating disorder (anorexia, bulimia, etc; screening will be carried out using the
  Diagnostic and Statistical Manual of Mental Disorders, fifth edition, if indicated)
- Malnourished patients (screening using the Malnutrition Universal Screening Tool, if indicated)
- BMI greater than 40 kg/m^2^
- Presenting gout
- Pregnant, planning pregnancy, or breastfeeding
- Use of diuretics at the time of sampling
- Difficulty or inconvenience in changing dietary habits and following the Mediterranean diet (allergies, food intolerances, and special diets)
- Participation in a clinical trial with drugs or dietary intervention in the year prior to inclusion in this study

#### Sample Size

There are no previous studies specifically evaluating the impact of MedDiet on skin involvement in patients with psoriasis. The only dietary intervention study in psoriasis to date is by Castaldo et al [8], which reported a decrease in Psoriasis Area and Severity Index (PASI) of 7.2 (95% CI –8.7 to –5.6) in a sample of 37 patients. However, this study differs significantly from MEDIPSO in design; it involved a single group without a control group, used a highly aggressive intervention including prolonged fasting periods, targeted only patients with obesity who have severe psoriasis, and excluded other adjuvant treatments, with a longer follow-up period.

In contrast, our study includes a control group, applies a less aggressive dietary intervention, and focuses on patients with mild-to-moderate psoriasis who are allowed to continue their stable topical treatments. Consequently, we anticipate the SD in PASI reduction at week 16 to be much lower, around 1. To detect a difference of 1 unit on the PASI scale, with an α risk of 0.05 and a  β risk of less than 0.2 in a bilateral contrast, we estimate that 19 participants per group (intervention and control) are needed. We assume a common SD of 1 and estimate a loss-to-follow-up rate of 15%.

#### Randomization

After signing the informed consent, all participants will be enrolled and randomly assigned into either the MedDiet intervention group or the standard-of-care group using closed envelopes. Randomization will be conducted using a computer-generated list created by an independent statistician, who is blinded to the trial and not involved in participant recruitment. The allocation will follow a 1:1 ratio. The recruiter will only open the envelope and learn of each patient’s group assignment after the informed consent form has been signed by the patient.

#### Blinding

The study is single-blinded, as only the investigator evaluating the PASI is blinded. Both the patient, the nutritionists responsible for carrying out the intervention, and the person in charge of follow-up and nonsubjective data collection are aware of the group to which the patient has been assigned.

#### Roles

The entire dermatology department will be involved in patient recruitment. The principal investigator will oversee initial screening, follow-up, data collection, and the evaluation of side effects. Randomization will be conducted using a sealed-envelope system, with the principal investigator opening the envelopes only after the patient has been enrolled in the study and the informed consent has been signed. This investigator will not be blinded.

The nutritional intervention was fully designed by nutritionists from the University of Navarra, who will implement the intervention with the support of the principal investigator. The nutritionists will also not be blinded.

Evaluation of nonobjective parameters, such as PASI, and data analysis will be conducted by coinvestigators who are blinded to the group assignments.

#### The Nutritional Intervention

The objective of the nutritional intervention in the MEDIPSO study is to enhance adherence to the MedDiet. A hybrid approach combining remote and face-to-face interactions will be implemented. During the initial visit, when the first blood sample is taken and the patient’s data are collected, initial dietary recommendations will be provided along with supportive materials, including instructions on the MedDiet, infographics, sample menus, and recipes. Following this, dietitian-nutritionists with experience from previous MedDiet intervention studies, such as PREDIMED, will conduct monthly telephone consultations with each participant.

During these consultations, the dietitian-nutritionists will assess adherence to the MedDiet and set individualized goals for improvement based on the participant’s needs. In addition to the printed materials provided at the first visit, participants will have the opportunity to discuss any questions or concerns with the research team.

Participants in the intervention group will receive 500 mL of EVOO each week, distributed during clinic visits. The goal is to encourage participants to consume at least 4 tablespoons of EVOO daily, using it as the primary culinary fat in their homes as part of the MedDiet.

The dietary recommendations for the intervention will align with the habits defined in the MedDiet according to the Energy-Restricted Mediterranean Diet Adherence Screener (er-MEDAS) scale (Table 1) [17]. Participants will be instructed to follow specific guidelines, including consuming 4 or more tablespoons of EVOO daily for cooking and seasoning; 2 or more servings (200 g per serving) of vegetables per day (with at least 1 serving being raw); 3 or more servings (125 g per serving) of fruits per day (including natural juices); 3 or more servings (60-80 g per serving) of legumes per week; 3 or more servings (150 g per serving) of fish or seafood per week (including at least 1 serving of oily fish); and 3 or more servings (30 g per serving) of nuts per week. Participants will be advised to choose white meats (eg, skinless poultry and rabbit) over red meat (eg, beef and pork) or processed meats (eg, sausages and hamburgers). Regular use of sofrito (a sauce made with chopped tomato, garlic, and onion simmered in olive oil) for cooking, at least twice per week, will also be encouraged. In addition, whole grains (eg, bread, pasta, and rice) should be chosen over refined cereals. The dietary plan will recommend eliminating or limiting the consumption of cream, butter, margarine, carbonated and sugary drinks, baked goods (eg, sweet desserts, cakes, pastries, and cookies), and ultraprocessed foods. The nutritional intervention will not prescribe a specific caloric intake or macronutrient distribution.

**Table 1:** Energy-Restricted Mediterranean Diet Adherence Screener scale score (the maximum possible score is 17; this was the scale used to assess the adherence to the Mediterranean diet during the study; a score ≤7 was considered to be poor adherence to Mediterranean diet).

Question to be asked to the patient	Criterion to score 1 point; otherwise, 0 is recorded
Do you use extra virgin olive oil as the principal source of fat for cooking?	Yes
How many servings of vegetables do you consume per day? Count garnish and side servings as half a serving; a full serving is 200 g.	≥2
How many pieces of fruit (including fresh-squeezed juice) do you consume per day?	≥3
How many servings of red meat, hamburger, or sausages do you consume per week? A full serving is 100-150 g.	≤1
How many servings (12 g) of butter, margarine, or cream do you consume per week?	<1
How many carbonated and sugar-sweetened beverages do you consume per week?	<1
How many servings (150 g) of pulses do you consume per week?	≥3
How many servings of fish and seafood do you consume per week? (100-150 g of fish, 4-5 pieces, or 200 g of seafood)	≥3
How many times do you consume pastry such as cookies, cake, or sweets per week?	<3
How many times do you consume nuts per week? (1 serving=30 g) per week?	≥3
Do you prefer to eat chicken, turkey, or rabbit instead of beef, pork, hamburgers, or sausages?	Yes
How many times a week do you eat cooked vegetables, pasta, rice, or other dishes dressed with sofrito (a tomato, garlic, onion, or leek sauce simmered with olive oil)?	≥2
Do you add sugar to your beverages (coffee, tea)?	No
How many servings of white bread do you consume per day (1 serving=75 g)?	<1
How many servings of whole-grain bread, pasta, or rice do you consume per week?	≥5
How many servings of refined bread, rice, and pasta do you consume per week?	<3
Do you drink wine? How much do you consume per week? (1 cup=100 mL)	Male 14-21 cups; female 7-14 cups

#### Study Protocol

The intervention period will last 16 weeks, comprising 3 face-to-face visits for all participants and 2 additional telephone visits for those in the intervention group.

During the baseline visit (visit 1), patients will sign the informed consent, undergo randomization, and have clinical information collected. A complete physical examination will be conducted, and blood samples will be obtained. For patients randomized to the intervention group, this visit will also include the provision of informational materials, the distribution of EVOO, and the initiation of the nutritional intervention.

At week 8, a face-to-face visit (visit 2) will be conducted. The primary objective of this visit is to perform a clinical evaluation to determine if the patient has experienced any clinical worsening that would necessitate the initiation of systemic treatment. If systemic treatment is deemed necessary, the corresponding analytical tests and vaccination protocol will be initiated. This visit at week 8 ensures that participation in the study does not delay the start of systemic treatment if required.

During the final visit (visit 3), clinical information will once again be collected, a complete physical examination will be performed, and final blood samples will be drawn. In addition, feedback will be gathered from the patient regarding their experience in the study, any challenges encountered, and suggestions for improvement. This feedback will be collected through an anonymous, free-response online survey, as well as a brief interview in which the patient can discuss any issues identified during the study with the investigator.

**Figure 1:**
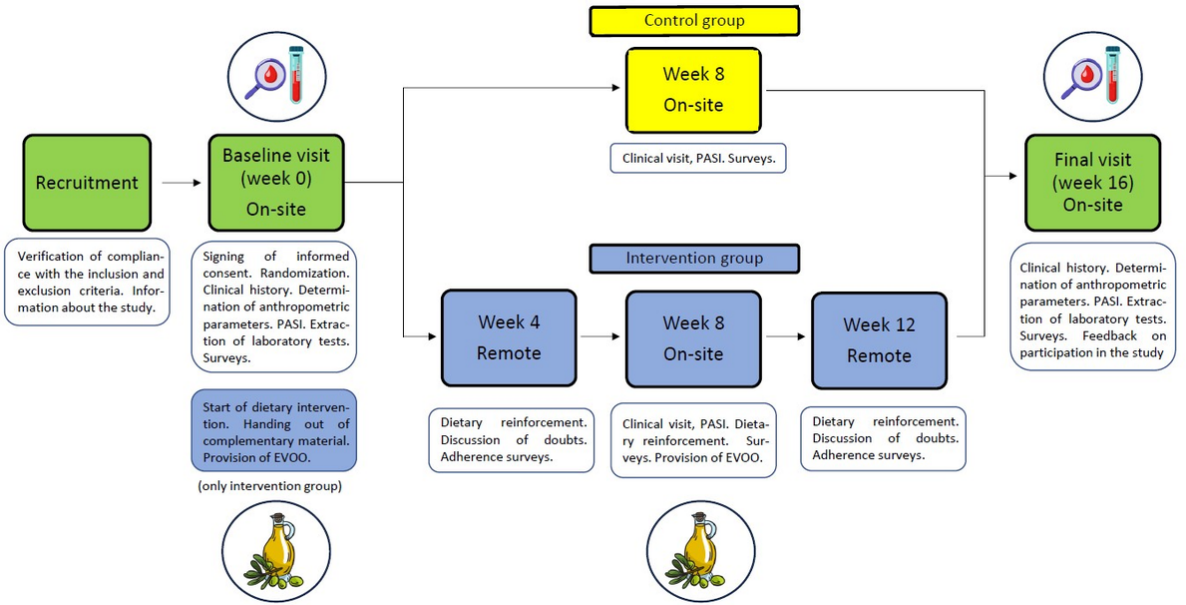
Comprehensive overview of the study protocol. As shown, patients in the control group will attend 3 on-site study visits. In contrast, patients in the Mediterranean diet intervention group will attend 3 on-site visits as well as 2 additional remote visits. Participants in the intervention group will receive 4 L of EVOO at weeks 0 and 8 (500 mL per week). Blood tests will be conducted at week 0 and week 16 in both groups. EVOO: extra virgin olive oil; PASI: Psoriasis Area and Severity Index.

The study protocol is summarized in Figure 1.

#### Outcomes

The primary outcome is the change from baseline in the PASI at week 16. PASI is the most widely used scale for assessing psoriasis severity and informing therapeutic decisions, with scores ranging from 0 (no lesions) to 72 (severe lesions across the entire body surface) [18].

The secondary outcomes include:

1. Change from baseline in adherence to the MedDiet at week 16: Adherence will be assessed using the validated er-MEDAS questionnaire, which scores from 0 to 17, with higher scores indicating better adherence [17].
2. Change from baseline in anthropometric parameters at week 16: This includes weight, BMI, and abdominal circumference, measured before and after the intervention.
3. Change from baseline in serum inflammatory interleukins at week 16: Levels of various ILs and cytokines will be assessed, including granulocyte-macrophage colony-stimulating factor, interferon gamma (IFNγ), IL-1β, IL 2, IL 4, IL 5, IL 6, IL 9, IL 10, IL 12 (p70), IL 13, IL 15, IL 17A/CTLA8, IL-17E/ IL-25, IL 17F, IL 21, IL 22, IL 23, IL 27, IL 28A/IFNλ2, IL 31, IL 33/NF HEV (mature), MIP-3α/CCL20, tumoral necrosis factor alpha (TNFα), and TNFβ/Lymphotoxin-α (LTA).
4. Change from baseline in metabolic blood parameters at week 16: changes in serum cholesterol, low-density lipoproteins, high-density lipoproteins, lipoprotein A, apolipoprotein A1, apolipoprotein B, fasting serum insulin, hemoglobin, A_1c_ and C-reactive protein (CRP) will be measured.
5. Change from baseline in the impact of the disease on patient’s life at week 16: quality of life will be assessed using the validated questionnaire Dermatology Life Quality Index [18]. Change in sleep quality will be evaluated using the Insomnia Severity Index [19] and emotional state changes will be measured using the Hospital Anxiety and Depression Scale [20].

### Data Analysis

Once data collection is complete, statistical analysis will be performed using StataIC 17 (Stata Corp). A descriptive analysis of the baseline demographic and clinical characteristics of the patients included in the trial will be conducted. Normality will be assessed using skewness, kurtosis, and histogram plots. Parametric variables will be reported as mean (SD), nonparametric variables as median (IQR), and categorical variables as n (%).

To evaluate differences between the intervention groups, statistical significance will be assessed using the Student *t* test for comparing 2 groups and the ANOVA test for comparisons involving multiple groups, for parametric variables. For nonparametric variables, the Wilcoxon rank-sum test and Kruskal-Wallis test will be applied. Pearson chi-square test will be used for categorical variables. An intention-to-treat analysis will be performed.

Linear mixed-effects models will be used to assess changes in nutritional variables from baseline to follow-up visits. Statistical significance will be determined using a 2-tailed level of significance, with *P* values less than .05 considered significant.

### Ethical Considerations

The study protocol (version 2.0; July 10, 2023) was approved by the Institutional Review Board of the Hospital Ramón y Cajal (Madrid) in July 2023 (170/23).

All personal and clinical information of potential and enrolled participants will be coded and used exclusively for clinical trial purposes. This information will be securely stored in the BioeBank 3.01.0.R26 storage system. Access to this data will be restricted to the investigators involved in this study. No monetary compensation was provided to the participants.

## Results

**Figure 2:**
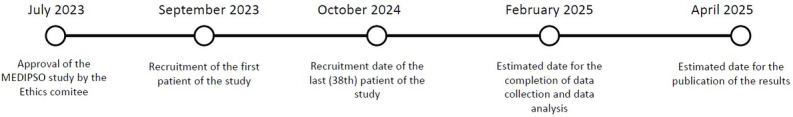
Estimated study timeline. MEDIPSO: Impact of the Mediterranean Diet on Patients with Psoriasis.

Enrollment concluded in October 2024, with data collection set to finish by February 2025. The findings will be presented at national and international conferences and published in peer-reviewed journals. The estimated study timeline is illustrated in Figure 2.

## Discussion

### Expected Findings

The relationship between psoriasis and the MedDiet has been explored in several cross-sectional studies. Barrea et al [21] conducted a case-control study involving 62 patients with untreated moderate-to-severe psoriasis and 62 healthy controls matched for age, sex, and BMI. This study found that patients with psoriasis were less adherent to the MedDiet than healthy controls. In addition, the consumption of EVOO and fish were identified as predictors of a lower PASI [21]. Similarly, Korovesi et al [22], in a study with a comparable design, reached analogous conclusions and also highlighted the beneficial effect of legume consumption on PASI reduction. Furthermore, Molina-Leyva et al [23] observed a lower severity of psoriasis in patients with greater adherence to the MedDiet, along with a reduction in CRP, a marker of systemic inflammation.

Despite the growing evidence suggesting the potential benefits of the MedDiet in psoriasis, the current evidence remains weak, primarily limited to nonexperimental studies [10]. In an attempt to provide more robust data, Castaldo et al [8] designed an experimental study where an aggressive weight loss was induced through a ketogenic diet followed by a hypocaloric Mediterranean-type diet. Significant reductions in skin involvement were observed, as measured by PASI. Interestingly, no linear correlation was found between weight loss and PASI, suggesting that ketone bodies and other dietary components from both dietary patterns may contribute to the observed anti-inflammatory effects [8]. However, the study’s design lacked a control group, making it difficult to attribute the anti-inflammatory effects solely to the dietary intervention. In addition, the study only included obese patients, leaving unanswered the critical question of whether these potential benefits of the MedDiet could also be observed in patients with normal weight.

The molecular mechanisms underlying the potential benefits of the MedDiet on psoriasis severity are not yet well characterized. Beyond psoriasis, greater adherence to the MedDiet has been shown to reduce the severity of other immune-mediated diseases, such as rheumatoid arthritis [24] and inflammatory bowel disease [25]. This observation has shifted attention toward the MedDiet’s potential systemic anti-inflammatory effects. Olive oil, a key component of the MedDiet, is rich in monounsaturated fatty acids. Virgin olive oil, in particular, retains all the lipophilic components of the olive, as well as α-tocopherol and phenolic compounds—molecules with strong antioxidant properties—whereas refined olive oil loses most of its antioxidants during the refining process [26]. Supporting this hypothesis, Mena et al [27] conducted a clinical trial demonstrating that patients who followed a MedDiet supplemented with EVOO exhibited a down-regulation of proinflammatory biomarkers associated with atherogenesis, including serum IL-6, soluble intercellular adhesion molecule-1, and CRP [27].

Our study’s prospective and longitudinal design offers a valuable opportunity to assess how different inflammatory molecules vary following a dietary intervention specifically in psoriasis, potentially illuminating the molecular mechanisms by which the MedDiet exerts its effects in immune-mediated diseases. A key strength and distinguishing factor of this study compared with previous research is the inclusion of a control group, which enhances the ability to attribute observed changes directly to the dietary intervention. This is particularly important given the frequent use of topical treatments in psoriasis patients, regardless of systemic therapy, and the episodic nature of psoriasis, where spontaneous improvements in severity can occur. In addition, maintaining close contact with a small cohort of patients will enable a thorough evaluation of logistical challenges, which will be crucial for planning future larger-scale multicenter trials.

Several limitations of the study design should be noted. First, this is a unicentric clinical trial, which may limit the external validity of the results. In addition, the interventional nature of the study—characterized by close follow-up and the free provision of EVOO—may mean that the findings are not generalizable to all patients with psoriasis. Furthermore, the MedDiet intervention and follow-up period is limited to 4 months, and the sample size is relatively small, potentially resulting in insufficient statistical power for some analyses. However, these limitations should be viewed in the context of the study’s proof-of-concept nature.

### Strengths and Limitations of This Study

First, there are no firm dietary recommendations that can be provided to patients with psoriasis. This study is a pioneering effort in implementing the MedDiet in this patient population, drawing on the expertise of epidemiologists and nutritionists who have specialized in MedDiet interventions, particularly in prestigious studies like the PREDIMED clinical trial.

Second, multiple outcomes resulting from the intervention such as psoriasis severity, cardiometabolic parameters, inflammatory cytokines, and quality of life will be evaluated. In addition, this clinical trial is designed to pave the way for a larger study, involving more patients, multiple centers, and extended follow-up periods.

Third, a possible limitation of this study is the relatively small sample size and the intervention duration of only 4 months, which reflects the proof-of-concept nature of the study.

Finally, the results of the nutritional intervention may not be generalizable to all patients with psoriasis for 2 reasons. First, the study population consists of patients with mild-to-moderate psoriasis who attend dermatology clinics, potentially indicating a higher level of health engagement than the general population. Second, while the provision of EVOO is a strength of the study, it may present a barrier in real-world situations due to the high cost of this product.

In conclusion, the MEDIPSO study was designed to generate high-quality evidence on the relationship between the MedDiet and psoriasis. Through this clinical trial, we aim to explore the anti-inflammatory effects of the MedDiet and their manifestation at both the clinical and molecular levels. In addition, this study is intended to lay the groundwork for future research in this area.

## Abbreviations

CRP: C-reactive protein
er-MEDAS: Energy-Restricted Mediterranean Diet Adherence Screener
EVOO: extra virgin olive oil
IFNγ: interferon gamma
IL: interleukin
MedDiet: Mediterranean diet
MEDIPSO: Impact of the Mediterranean Diet on Patients with Psoriasis
PASI: Psoriasis Area and Severity Index
PREDIMAR: Prevention of Recurrent Arrhythmias with Mediterranean Diet
PREDIMED: Prevention with Mediterranean Diet

## Data Availability

Access to the full protocol, participant-level dataset, and statistical code can be granted upon reasonable request to the investigative team.
